# Supplementary material for: Comparative Diversity and Functional Traits of Fungal Endophytes in Response to Elevated Mineral Content in a Mangrove Ecosystem
Source: J Fungi (Basel). 2023 Dec 11;9(12):1186. doi: 10.3390/jof9121186 (PMC10744541; doi:10.3390/jof9121186)
Supplement: Supplementary file 1 [file jof-09-01186-s001.zip › jof-2753663-supplementary.pdf]

Table S1. Morphological descriptions of the different morsphospecies of mangrove-associated fungi

Taxa	No. of specimens	Image	Characteristics
***Alternaria alternata* (Fr.)**	1	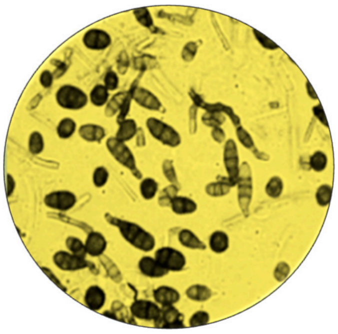	Thin to thick colony, greyish to black, and velvety in texture. Conidia is oblclavate to ellipsoidal with pale to olivaceous color.
***Aspergillus terreus* Thom.**	4	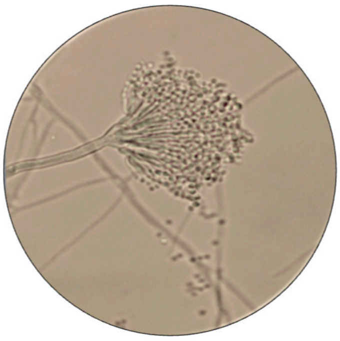	Colony is floccose and thick; white, brownish to cinnamon in color; powdery and velvety. Conidia are pale, hyaline, spherical to globose.
***Aspergillus parasiticus* Speare**	1	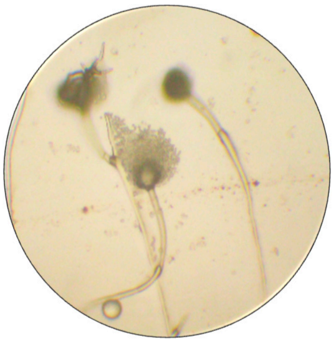	Colony is white, concentric deep green; cottony. Spores are pale hyaline to light brown and globose
***Aspergillus* sp.**	1	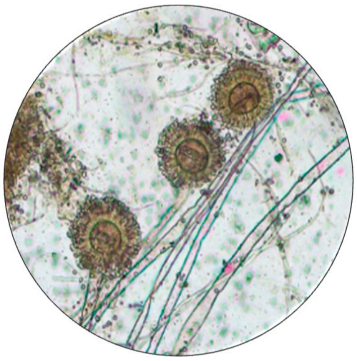	Colony is floccose, dark green with white edges; cottony. Conidia is hyaline and spherical in shape.
***Cladophialophora* sp1.**	15	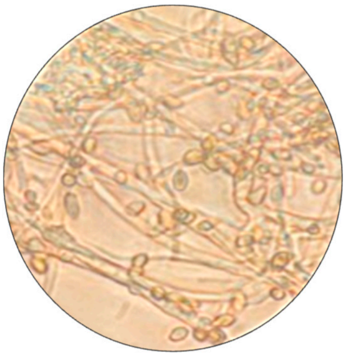	Thick, olivaceous green to olive colored colony with velvety texture. Pale olivaceous green to brown. Conidia is ellipsoidal to spindle-shaped

***Cladophialophora* sp.2**	1	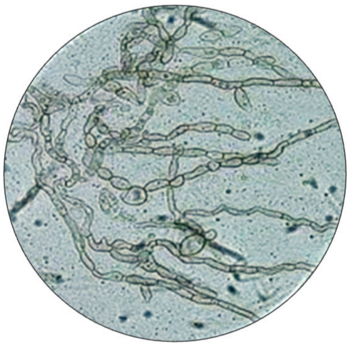	Floccose, colonies were characterized by their color, which ranged from olivaceous-green to grey, The conidia themselves were hyaline, smooth, and thin-walled, with shapes ranging from ovoid to ellipsoidal, and they were not segmented by septa.
***Cladosporium* sp.1**	2	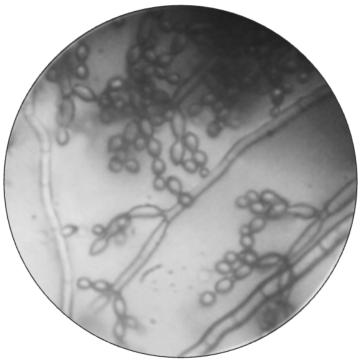	The colonies displayed colors such as olivaceous grey, whitish, or pale olivaceous and exhibited a luxurious growth of mycelium conidia were catenate, forming branched chains.
***Cladosporium* sp.2**	1	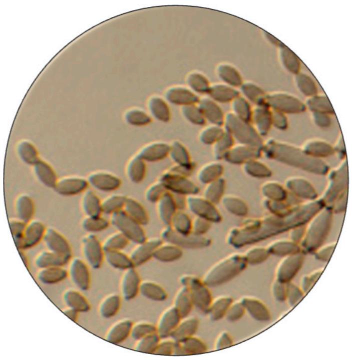	The colony transforms in color from a dark, dull green to a deep green shade. It possesses a velvety or cushiony texture and features a pale green margin with a white to clear boundary. conidiophore is distinct from the hyphae, showing branching and several enlargements with brown or olivaceous brown color. Conidia form in chains, branching out and have a cylindrical to ellipsoidal shape.
***Cladosporium cladosporioides* de Vries.**	6	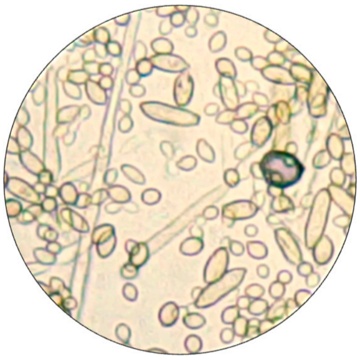	The mycelium was immersed, sparsely branched, or sparingly branched, pale olivaceous brown to pale brown. The conidia took on shapes such as sub-globose, obovoid, and limoniform, and they lacked any septation.

***Cladosporium herbarum* (Pers.) Link.**	1	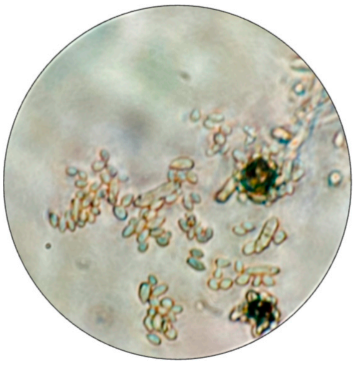	The colony had a regular, undivided margin that appeared white, and the reverse side had a velvety texture. Their shapes included ellipsoid, limoniform, and occasionally fusiform, and htey were primarily non-septate.
***Cladosporium macrocarpum* Preuss**	2	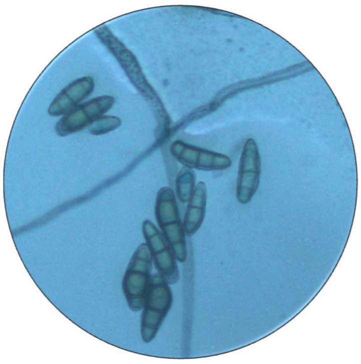	The colony undergoes a color change from a dark, dull green to a dark gray with an olivaceous gray hue. Their shapes can be oval, lemon-shaped, or obovoid.
***Diaporthe* sp.**	9	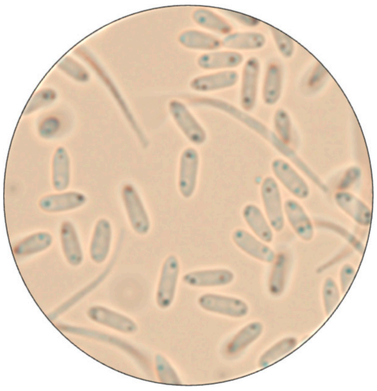	The colony exhibits a color spectrum ranging from white to a yellowish hue. The conidia are clear (hyaline), lacking septa,
***Exophiala* sp.**	1	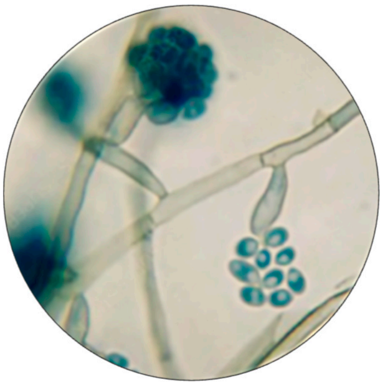	Velvety and cottony appearance colony. The conidia themselves are ellipsoidal, consist of a single cell, and are typically found in clusters.

***Fusarium proliferatum* Gerlack& Nirenberg.**	5	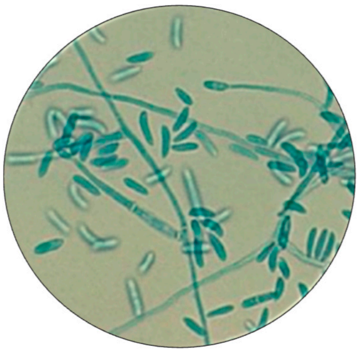	The colony has a fluffy and floccose texture with visible aerial mycelia. The macroconidia are elongated and initially straight but can also have a curved, sickle-like shape. The microconidia are shaped like a club (clavate) or obovoid. There are no chlamydospores present.
***Fusarium solani* Sacc.**	9	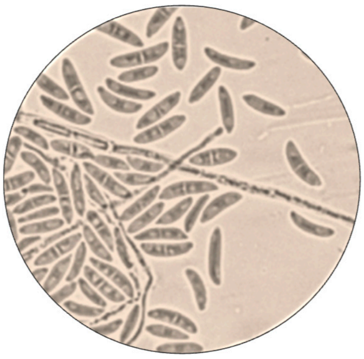	mycelia exhibited a color range from white to pale cream. The macroconidia are long, straight, and have five distinct segments or partitions. Microconidia are smaller spores that are abundant and come in various shapes, ranging from oval to spherical.
***Leptosphaeria* sp.**	1	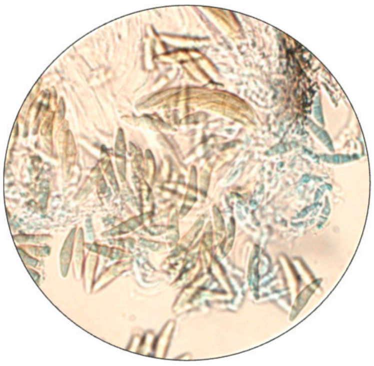	Irregular, velvety mass with the presence of small, grain-like white hyphae. The conidia are one-celled and transparent, typically having partitions, and they are mostly curved in shape.
***Lophiostoma* sp.**	5	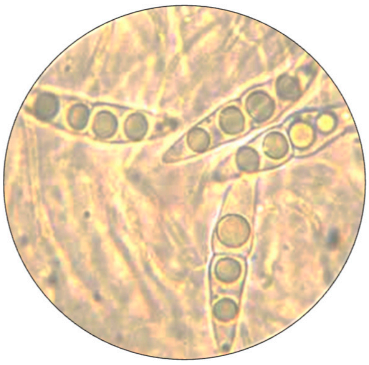	Velvety and dense; filamentous when matured. Distinct presence of numerous pseudoparaphyses, wide, septate, hyaline, branched and anastomosed (not observed in the sample). Ascus is fissitunicate and clavate, with four-cell ascospores; bi-seriate and constricted

Neopestalotiopsis clavispora **Maharachch, Hyde & Crous.**	2	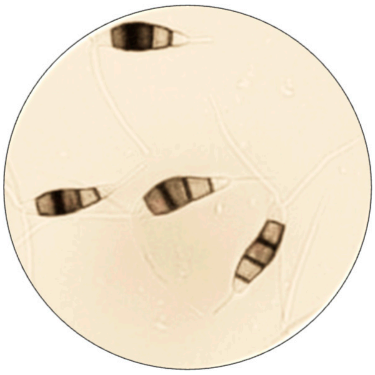	manifests as a white, cottony, and fluffy texture with undulating edges, featuring a rich and dense mycelial layer on the surface. The conidia have a shape that ranges from spindle-shaped to ellipsoidal, with a slight curve, and are composed of five cells.
Neopestalotiopsis egyptiaca **Ismail, Perrone & Crous.**	4	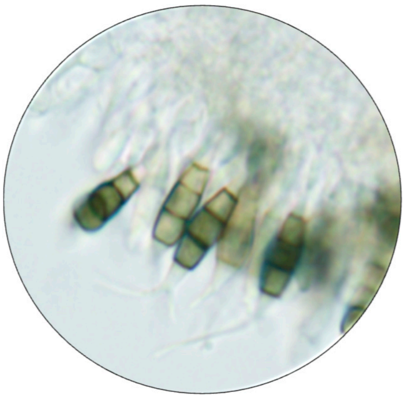	Smooth to slightly undulating edge and a somewhat raised white appearance. The conidia are smooth and have a subtle curve, being slightly narrower than those of *N. clavispora.*
Neopestalotiopsis rhizophorae **Norphanphoun, Wen & Hyde**	11	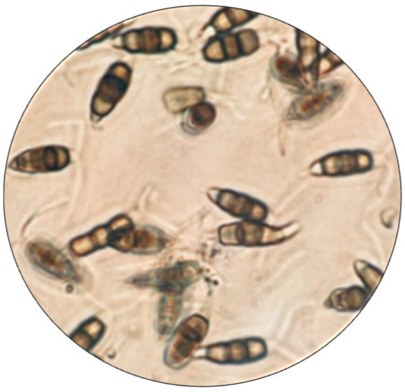	Filamentous colony having a smooth to slightly undulating edge and a somewhat raised and fluffy white appearance. The conidia can sometimes appear rough, although they are usually smooth.
Neopestalotiopsis **sp.1**	5	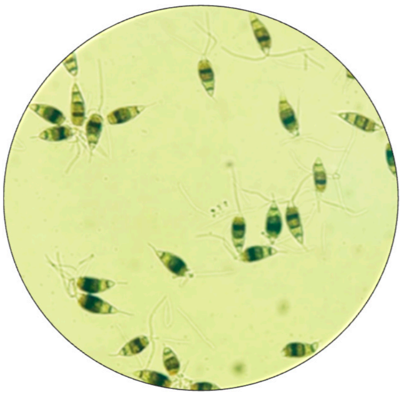	Filamentous colony having a smooth to slightly undulating edge and a somewhat raised and fluffy white appearance. The conidia are fusiform to clavate and mostly straight to slightly curved, thin and smooth-walled, made up of four -five segments, with the upper three cells having a darker color and the lower median cell being pale

*Nigrospora oryzae (***Berk. & Broome) Petch.**	2	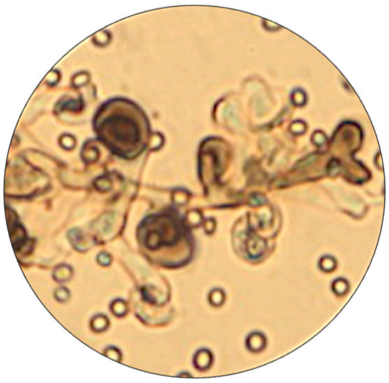	dense, irregular, convex, and typically pale, olivaceous grey appearance. The conidia can vary in color, ranging from dark brown to pale brown, and they have a shape that is either sub-globose or globose.
Penicillium oxalicum **Currie & Thom.**	9	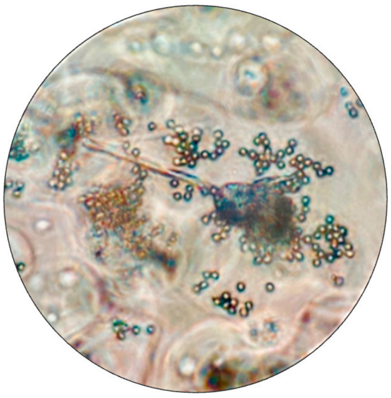	glossy appearance which turns velvety or leathery as it matures, and it featurees a white creamy margin. The conidiophores have terminal branching and typically possess 2-3 metulae. In the observed sample, they are arranged in a single vertical row, which is referred to as mono-verticilliate.
Pestalotiopsis **sp.1**	5	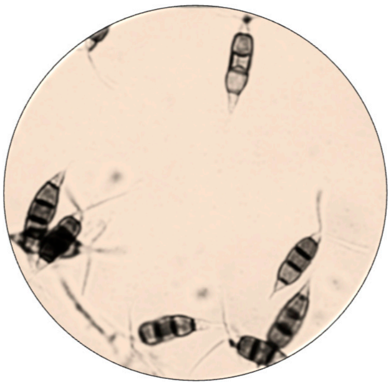	Mycelia exhibit a white, cottony, and fluffy appearance. The conidia consist of five cells, and their shape can vary, ranging from straight to curved.
Pestalotiopsis microspora **(Speg.) Zhao & Nan Li**	5	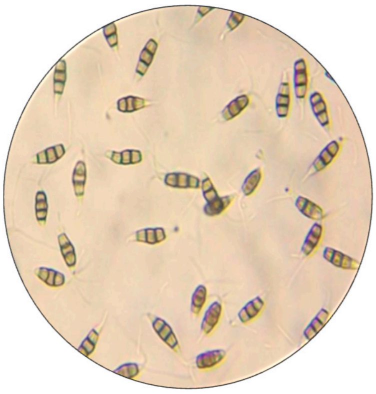	mycelia exhibit a white, cottony, and fluffy appearance with a flower-like pattern. The conidia consist of five cells, and their shape can vary, ranging from straight to curved, broad and 5-celled

Pestalotiopsis protearum **Maharachch, Hyde & Crous**.	5	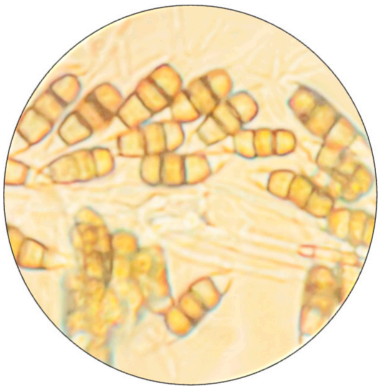	Greyish or off-white color mycelia. The conidia consist of four cells, and their shape can vary, ranging from straight to curved, broad. They may appear ellipsoidal or obovoid without constriction at the septa. Color is the same all throughout the conidia, the basal cell is usually paler, typically in shades of brown to dark brown.
Pestalotiopsis **sp.2**	2	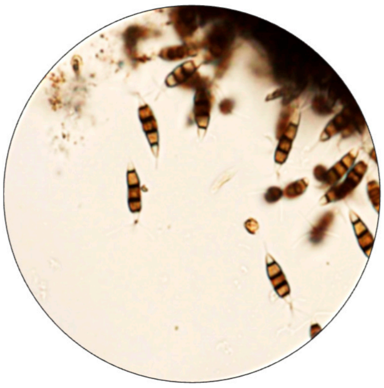	White, cottony, and fluffy appearance with undulated margin. The conidia consist of four or five cells, and their shape can vary, ranging from straight to curved, narrow or broad. They may appear ellipsoidal or obovoid with or without constriction at the septa
Phaeosphaeriopsis **sp**.	1	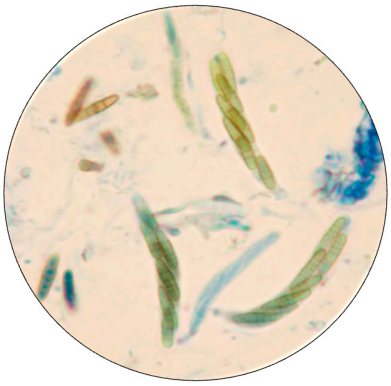	It begins with a white color but matures into shades of brown or grey. The asci are 8-spored and have a two-layered structure, with a cylindrical to club-shaped appearance
Phoma glomerata **Chen & Cai**	6	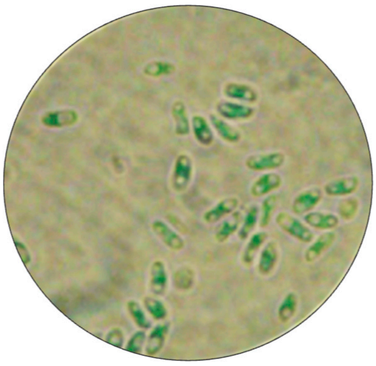	Colonies are typically characterized by their velvety texture, can range from powdery to woolly in appearance. The conidia consist of a single cell, while chlamydospores are multicellular and appear in plentiful chains

***Phoma* sp.1**	4	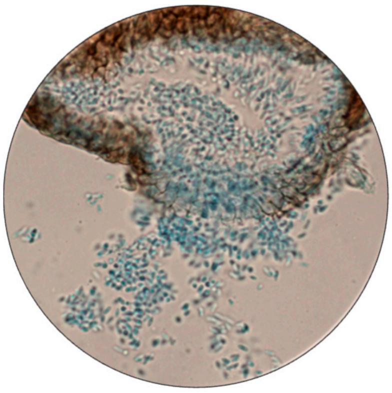	Grey to black in color. These colonies are black, olivaceous, or even grey in color. The conidia consist of a single cell while chlamydospores are multicellular and appear in plentiful chains
***Phyllosticta* sp**.	2	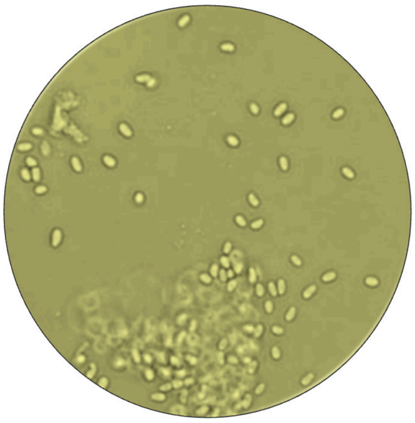	They can appear either powdery or velvety in texture. The color of these colonies ranges from greyish to dark olive green. The conidiophores have a cylindrical or flask-like shape. The conidia are transparent, without partitions, and have forms that are ovoid, ellipsoidal, or somewhat round.
***Pseudopestalotiopsis curvatispora* Norphanphoun, Wen & Hyde**	5	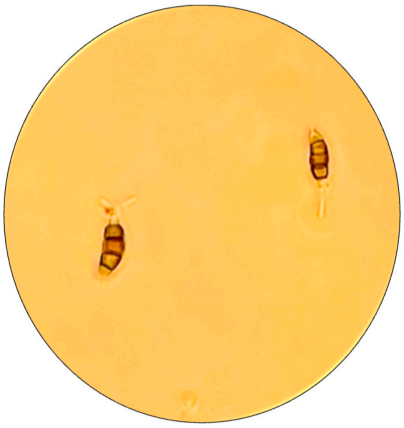	Filamentous to circular, medium dense, aerial mycelium, mostly raised with filiform margin. The conidia have a spindle-shaped to club-shaped form, and they can be either straight or slightly curved
***Pseudopestalotiopsis* sp.**	9	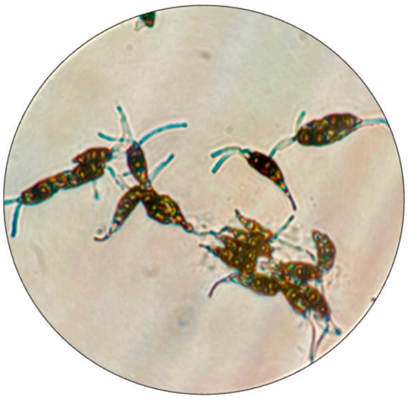	The colonies are filamentous to circular, medium dense, aerial mycelium, mostly raised with filiform margin. The conidia have a spindle-shaped to club-shaped form, and they can be either straight or slightly curved

Ramichloridium biverticilliatum **Arzanlou & Crous.**	3	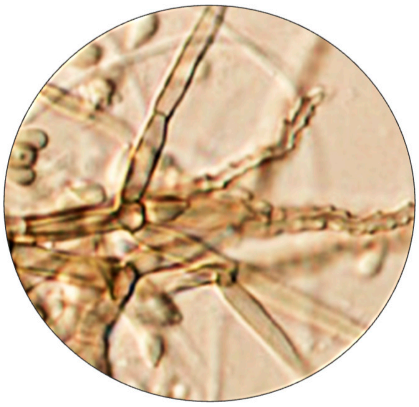	Filamentous to circular, medium dense, velvety and dry. Conidiophores are pale, brown, and profusely branched, biverticilliate with at least three-levels of main branches.
Schizophyllum commune **Fr.**	9	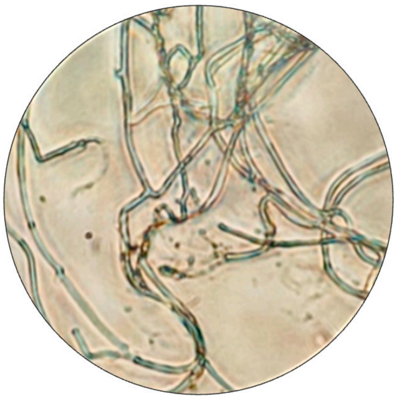	Mycelia is white to yellowish in color. Texture is woolly, cottony, and thick. Microscopic characters include hyaline hyphae, septate and non-dichotomously branching.
Talaromyces **sp.**	1	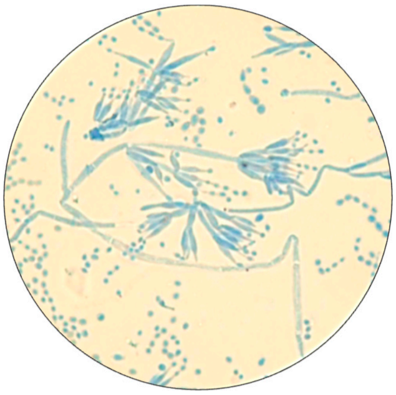	Mycelia is yellowish to greenish which becomes grey at maturity. Biverticillate conidiophores, hyaline and smooth-walled.
Ulocladium **sp.**	2	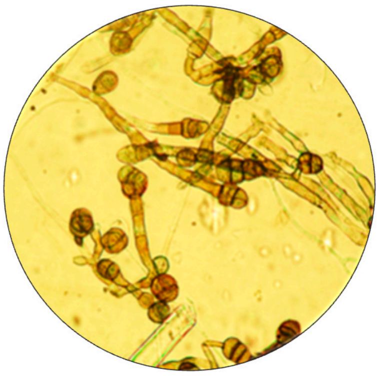	Mycelia is initially olivaceous then turns grey to light grey when mature. Erect, straight or flexuose conidiophores, which are typically unbranched.

Table S2. Detailed morphological description of the selected MFEs *Neopestalotiopsis*, *Pestalotiopsis*, and *Pseudopestalotiopsis*

Taxa	No. of isolates	Image	Colony morphology	Spore morphology
***Neopestalotiopsis clavispora* Maharachch, Hyde & Crous**	5	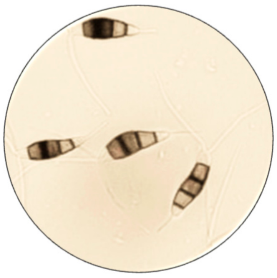	It displays rapid growth on a PDA medium. Initially, it manifests as a white, cottony, and fluffy texture with undulating edges, featuring a rich and dense mycelial layer on the surface. As it matures, you can observe scattered black conidiomata, which appear slimy and shiny on the Petri plate	The conidia have a shape that ranges from spindle-shaped to ellipsoidal, with a slight curve, and are composed of five cells. The middle cells have darker colors, while the lower median cells are pale and transparent. At the top of the conidia, you can find either two or three hairlike-structures, with a knob-like structures at the end, and there is a single appendage at the bottom of the conidia.
***Neopestalotiopsis egyptiaca* Ismail, Perrone & Crous.**	2	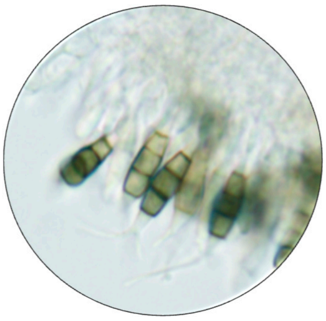	It exhibits rapid growth on a PDA medium, with a colony having a smooth to slightly undulating edge and a somewhat raised white appearance. There is some small aerial mycelium, and the conidiomata, mainly occurring individually and dispersed, take the form of slimy black masses as they develop.	The conidia are smooth and have a subtle curve, being slightly narrower than those of *N. clavispora.* They are made up of four segments, with the upper three cells having a darker color and the lower median cell being pale. The conidia can sometimes appear rough, although they are usually smooth.
***Neopestalotiopsis rhizophorae* Norphanphoun,Wen& Hyde.**	4	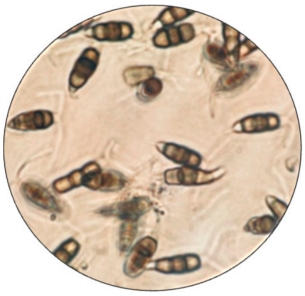	It exhibits rapid growth on a PDA medium, with a filamentous colony having a smooth to slightly undulating edge and a somewhat raised and fluffy white appearance. There is some small aerial mycelium, and the conidiomata, mainly occurring individually and dispersed, take the form of slimy black masses as they develop.	The conidia are fusiform to clavate and mostly straight. To slightly curved, thin, a nd smooth-walled, wider than *N. egyptiaca* but narrower than *N. clavispora*. They are made up of four segments, with the upper three cells having a darker color and the lower median cell being pale. The conidia can sometimes appear rough, although they are usually smooth. At the top of the conidia, there are 2-3 tubular, unbranched

				appendages, arranged at the center, and there are no knob-like structures.
***Neopestalotiopsis* sp.1**	11	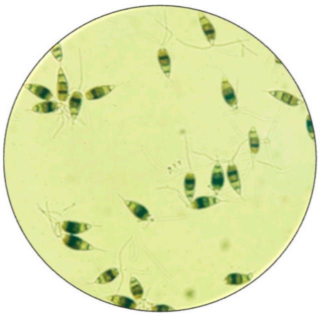	It exhibits rapid growth on a PDA medium, with a filamentous colony having a smooth to slightly undulating edge and a somewhat raised and fluffy white appearance. Usually, conidiomata arises from mycelia and produces black slimy and shiny cell mass.	The conidia are fusiform to clavate and mostly straight to slightly curved, thin and smooth-walled, made up of four -five segments, with the upper three cells having a darker color and the lower median cell being pale. At the top of the conidia, there are 2-3 tubular, unbranched appendages, arranged at the center, with or without a knob-like structure.
***Pestalotiopsis* sp.1**	9	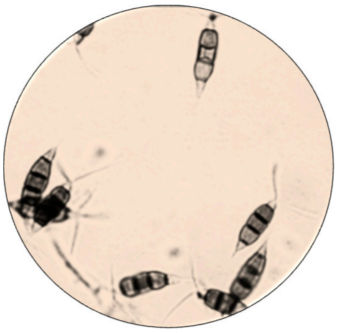	At the early stages, the mycelia exhibit a white, cottony, and fluffy appearance. However, as they mature, they transform into a greyish or off-white color. The colony maintains a smooth and continuous edge. Additionally, the reverse side of the colony appears brown to dark brown in color.	The conidia consist of five cells, and their shape can vary, ranging from straight to curved. They may appear ellipsoidal or fusiform and are constricted at the septa, with the second apical cell having a thicker septum. The concolorous cells have distinct colors, typically in shades of brown to dark brown. They are usually thin-walled and are numerous in appearance. At the apex, there are short appendages with knob-like structures, with typically 2-3 appendages present.
***Pestalotiopsis microspore* (Speg.) Zhao & Nan Li**	5	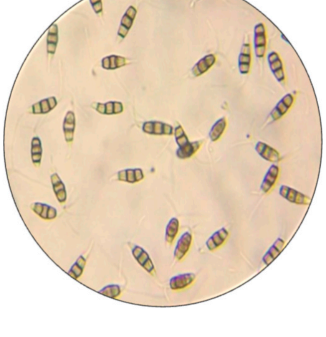	At the early stages, the mycelia exhibit a white, cottony, and fluffy appearance with a flower-like pattern. However, as they mature, they transform into a greyish or off-white color. Additionally, the reverse side of the colony appears brown to dark brown in color.	The conidia consist of five cells, and their shape can vary, ranging from straight to curved, broad and 5-celled. They may appear ellipsoidal or fusiform and are constricted at the septa. The concolorous cells have distinct colors, typically in shades of brown to dark brown. They are usually thin-walled and

				are numerous in appearance. At the apex, there are short appendages with knob-like structures, with typically 2-3 appendages present.
***Pestalotiopsis protearum* Maharachch, Hyde & Crous**.	5	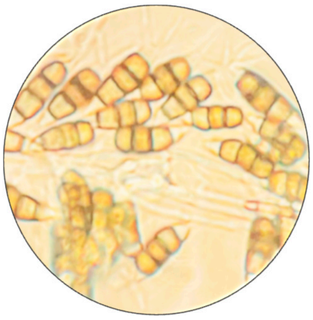	At the early stages, the mycelia exhibit a white, cottony, and fluffy appearance with a flower-like pattern. However, as they mature, they transform into a greyish or off-white color. Additionally, the reverse side of the colony appears brown to dark brown in color.	The conidia consist of four cells, and their shape can vary, ranging from straight to curved, broad. They may appear ellipsoidal or obovoid without constriction at the septa. Color is the same all throughout the conidia, the basal cell is usually paler, typically in shades of brown to dark brown. They are usually thin-walled and are numerous in appearance. At the apex, there are short appendages with knob-like structures, with typically 2-3 appendages present.
***Pestalotiopsis* sp.2**	2	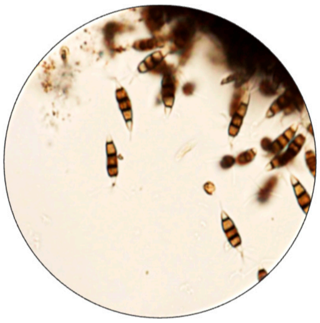	At the early stages, the mycelia exhibit a white, cottony, and fluffy appearance with undulated margin. Presence of whitish to pale acervuli was observed to most of the colonies. However, as they mature, they transform into a greyish or off-white color. Additionally, the reverse side of the colony appears brown to dark brown in color.	The conidia consist of four or five cells, and their shape can vary, ranging from straight to curved, narrow or broad. They may appear ellipsoidal or obovoid with or without constriction at the septa. Color is the same all throughout the conidia, the basal cell is usually paler, typically in shades of brown to dark brown. They are usually thin-walled and are numerous in appearance. At the apex, there are short appendages with knob-like structures, with typically 2-3 sometimes 4 appendages present.

Pseudopestalotiopsis curvatispora **Norphanphoun, Wen & Hyde**.	5	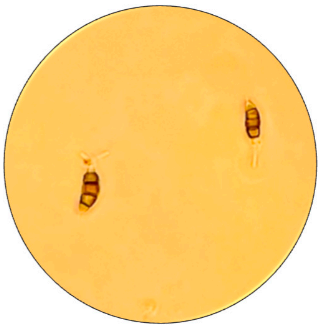	The colonies are filamentous to circular, medium dense, aerial mycelium, mostly raised with filiform margin, fluffy, white, and cottony in appearance. Appearance of slimy and shiny cell mass when matured.	The conidia have a spindle-shaped to club-shaped form, and they can be either straight or slightly curved. They consist of four segments and are either transparent or pale brown, with a consistent color throughout.
Pseudopestalotiopsis **sp.**	9	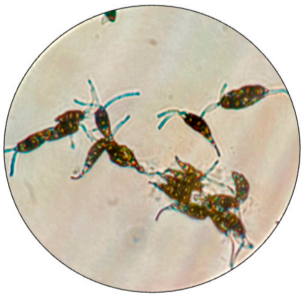	The colonies are filamentous to circular, medium dense, aerial mycelium, mostly raised with filiform margin, fluffy, white, and cottony in appearance. Appearance of slimy and shiny cell mass when matured.	The conidia have a spindle-shaped to club-shaped form, and they can be either straight or slightly curved. They consist of four segments and are either transparent or pale brown, with a consistent color throughout except that this strain is narrower than *Ps. curvatispora*.
